# Regional changes in tuberculosis disease burden among adolescents in South Africa (2005–2015)

**DOI:** 10.1371/journal.pone.0235206

**Published:** 2020-07-01

**Authors:** Erick Wekesa Bunyasi, Humphrey Mulenga, Angelique K. K. Luabeya, Justin Shenje, Simon C. Mendelsohn, Elisa Nemes, Michele Tameris, Robin Wood, Thomas J. Scriba, Mark Hatherill

**Affiliations:** 1 South African Tuberculosis Vaccine Initiative, Faculty of Health Sciences, University of Cape Town, Cape Town, South Africa; 2 Desmond Tutu HIV Center, Faculty of Health Sciences, University of Cape Town, Cape Town, South Africa; University of London, UNITED KINGDOM

## Abstract

**Background:**

Adolescents in the Western Cape Province of South Africa had high force of *Mycobacterium tuberculosis* (MTB) infection (14% per annum) and high TB incidence (710 per 100,000 person–years) in 2005. We describe subsequent temporal changes in adolescent TB disease notification rates for the decade 2005–2015.

**Method:**

We conducted an analysis of patient–level adolescent (age 10–19 years) TB disease data, obtained from an electronic TB register in the Breede Valley sub–district, Western Cape Province, South Africa, for 2005–2015. Numerators were annual TB notifications (HIV–related and HIV–unrelated); denominators were mid–year population estimates. Period averages of TB rates were obtained using time series modeling. Temporal trends in TB rates were explored using the Mann–Kendall test.

**Findings:**

The average adolescent TB disease notification rate was 477 per 100,000 for all TB patients (all–TB) and 361 per 100,000 for microbiologically–confirmed patients. The adolescent all–TB rate declined by 45% from 662 to 361 per 100,000 and the microbiologically–confirmed TB rate by 38% from 492 to 305 per 100,000 between 2005–2015, driven mainly by rapid decreases for the period 2005–2009. There was a statistically significant negative temporal trend in both all–TB (per 100,000) (declined by 48%; from 662 to 343; p = 0·028) and microbiologically confirmed TB (per 100,000) (declined by 49%; from 492 to 252; p = 0·027) for 2005–2009, which was not observed for the period 2009–2015 (rose 5%; from 343 to 361; p = 0·764 and rose 21%; from 252 to 305; p = 1·000, respectively).

**Interpretation:**

We observed an encouraging fall in adolescent TB disease rates between 2005–2009 with a subsequent plateau during 2010–2015, suggesting that additional interventions are needed to sustain initial advances in TB control.

## Introduction

Historical tuberculosis (TB) notification rates in the greater Cape Town region of South Africa have changed markedly over the last century. The annual TB disease rate was approximately 450 per 100,000 general population between 1910–1950, but decreased in parallel with introduction of TB chemotherapy to the 20^th^ century nadir of 250 per 100,000 in 1970,[1] followed by a sustained increase to 850 per 100,000 in 2005, driven primarily by the HIV epidemic.[1] The year 2005 was key for national HIV and TB control, due to scale–up of antiretroviral therapy (ART) in public health facilities, with increasing ART coverage over subsequent years.[2] It was hoped that introduction of the sputum Xpert MTB/RIF assay as the primary test for TB diagnosis in 2013 would improve diagnostic yield, laboratory efficiency, and ability to detect rifampicin–resistant *Mycobacterium tuberculosis* (MTB) faster than liquid culture.[3] Introduction of these measures against the background of sustained programmatic TB control, including direct observation of treatment (directly observed treatment, short–course; DOTS),[4] raises the question of whether these interventions had measurable impact on TB rates in South African communities. Demonstration of temporal changes in the epidemic is important for re–appraisal of regional TB control in line with global “End TB Strategy” targets.[5] Whereas early childhood (≤ 5 years) TB disease reflects recent MTB transmission, and adult (≥20 years) TB disease is a function of cumulative lifetime risk of infection and progression to disease, trends in adolescent (10–19 years) TB disease rates reflect medium–term changes in the epidemic and impact of TB control interventions.[6]

We aimed to describe temporal changes in adolescent TB notification rates over the decade 2005–2015, based on analysis of electronic health service data for the Breede Valley sub–district, a high TB incidence setting near Cape Town, South Africa. Background TB rates among adults and children are provided for context.

## Methods

### Study design, population and setting

This is an analysis of electronic TB notification data from health authorities in the Breede Valley subdistrict, Western Cape Province, South Africa, between 1^st^ January 2005 – 31^st^ December 2015. Estimated population in 2005 was 154,565, of whom 28,643 (18·5%) were adolescents. Average annual population growth between 2005–2015 was 1·31%.[7]The general population composition in this sub–district in 2010 was 63·3% Mixed Race, 24·3% Black African, 10·7% Caucasian, and 1·7% other classifications;[7] estimated general population HIV prevalence was 3·7% in 2005 and 4·6% in 2010.[8] In the local general population, the proportion of all people living with HIV and receiving antiretroviral therapy (ART) increased from <1% in 2005 to 12% in 2007, 40% in 2013 and 55% in 2015.[8] Sputum Xpert MTB/RIF was introduced in place of sputum smear microscopy as the primary TB diagnostic test from 2013 onwards, otherwise TB prevention and treatment guidelines did not change substantially between 2005–2015.[9] Over the period of the study, the standard of care in public healthcare facilities involved offering HIV counselling and testing to all individuals diagnosed with TB disease. Screening and confirmation of adolescent HIV diagnosis was via HIV rapid antibody tests. However, studies have reported gaps in TB and HIV testing, linkage to care and treatment.[10], [11]

### Definitions

TB disease notification was defined as an episode of TB disease that was electronically notified by public health authorities. All treatment for TB disease is undertaken in public health facilities and is free of charge. The regional TB control program maintains both a paper record and from 2001 onwards an electronic register of TB patients treated in health facilities. TB disease include all TB diagnoses, regardless of clinical site or diagnostic approach (microbiologically confirmed or clinically diagnosed), that were treated and notified by public health authorities, with the exclusion of patients started on treatment outside the study area and referred to facilities within the study area; and patients re–treated after loss to follow–up or treatment failure and re–registered, in line with WHO recommendations.[12], [13] Due to high rates of re–infection after cure, instances of relapse or re–infection were regarded as new TB events. People who received only isoniazid chemoprophylaxis were excluded.

Adolescents were defined as people aged 10–19 years[14], adults as people aged ≥ 20 years and children as people aged <10 years. Microbiologically–confirmed TB was defined as sputum smear microscopy positive for acid–fast bacilli and/or positive liquid MTB culture and/or positive Xpert MTB/RIF assay on one or more occasions. TB patients were defined as living with HIV if a positive HIV serology result was recorded, or if the patient was recorded to be receiving ART or co–trimoxazole prophylaxis. HIV–negative status was defined by a recorded HIV–negative serology result. All other patients were considered to be of unknown HIV status.

### Statistical analysis

TB notification rate was determined by dividing the annual number of notified TB patients by the estimated mid–year population, multiplied by 100,000. Annual percentage change in TB rate was obtained by dividing the difference between TB rate for a given year and TB rate for the previous year by the TB rate for the previous year multiplied by 100%. The annual mid–year population estimate was derived from the 2001 and 2011 national census[15] and adjusted for annual population growth assuming linear growth between census estimates.[7] Trend analysis for demographic and clinical characteristics was assessed using the Chi–squared test for trend (*X*^*2*^_trend_), except for age and TB rates for which the Mann–Kendall[16] test was used, allowing for quantification of the magnitude of trend using the Theil–Sen median slope estimator.[17] The Durbin–Watson statistic was used to test and quantify autocorrelation in TB rates. The average TB rate for each period was estimated using a first order Box–Jenkins autoregressive integrated moving–average (ARIMA). All statistical tests were 2–sided at alpha 0·05.

### Ethical approval

Ethical approval was obtained from the University of Cape Town Human Research Ethics Committee (HREC REF 163/2016). This study obtained anonymized electronic reports from public health authorities before analysis. Therefore, the institutional ethics committee waived the need for individual consent.

## Results

### Demographic and clinical characteristics

A total of 21,250 TB patients were notified in Breede Valley sub–district between 2005–2015, of whom 1,461 (6·9%) were adolescents. The annual number of adolescent TB patients ranged between 102 (6·3%)– 187 (7·9%), with a statistically significant negative trend in proportion of all patients, falling from 8% to 6% between 2005–2015. The median age of adolescent TB patients also increased by an average of 1·0 (0·6–1·5) month annually over that period (*p*<0·001) (Table 1). 50% of adolescent TB patients were between the age of 17–19 years. 77% of adolescent TB was microbiologically–confirmed and this proportion did not substantially change after Xpert MTB/RIF scale–up in 2013. HIV testing of adolescent TB patients markedly increased from 1% to 88% between 2005–2015, when 45% of adolescent TB patients were known to be HIV–infected (2015). The proportion of all TB occurring among children < 10 years of age statistically significantly increased (from 34% to 39%; p<0·001); those among adolescents statistically significantly reduced (from 8% to 6%; p = 0·008); whereas those among adults (≥20 years) statistically significantly reduced between 2005–2015 (from 58% to 55%; p = 0·043).

**Table 1:** Demographic and clinical characteristics of adolescent TB patients.

Variable	2005	2006	2007	2008	2009	2010	2011	2012	2013	2014	2015	P	2005–2015
Patients (Adolescents/All; %)	187/2372 (7·9%)	161/2155 (7·5%)	146/1963 (7·4%)	135/1788 (7·6%)	102/1631 (6·3%)	129/1821 (7·1%)	121/1927 (6·3%)	118/1761 (6·7%)	132/1798 (7·3%)	114/2047 (5·6%)	116/1987 (5·8%)	<0·001	1461/21250 (6·9%)
Age Median (IQR)	16·0 (14·0–18·0)	16·0 (14·0–18·0)	17·0 (15·0–18·0)	17·0 (15·9–18·6)	17·9 (15·9–18·7)	17·6 (15·3–19·1)	16·7 (13·8–18·6)	17·1 (15·0–18·9)	17·2 (14·9–18·7)	17·4 (15·1–18·9)	17·2 (15·4–18·5)	<0·001	17·0 (15·0–18·6)
Sex (Male; n/N; %)	88/187 (47·1%)	69/161 (42·9%)	66/146 (45·2%)	55/135 (40·7%)	45/102 (44·1%)	70/129 (54·3%)	65/118 (53·7%)	46/118 (39·0%)	73/132 (55·3%)	57/114 (50·0%)	59/116 (50·9%)	0·350	693/1461 (47·4%)
Proportion of adolescent TB microbiologically confirmed	139/187 (74·3%)	125/161 (77·6%)	117/146 (80·1%)	102/135 (75·6%)	75/102 (73·5%)	104/129 (80·6%)	92/121 (76·0%)	99/118 (83·9%)	97/132 (73·5%)	82/114 (71·9%)	98/116 (84·5%)	0·465	1130/1461 (77·3%)
Clinical type (PTB/(PTB+EPTB)	168/187 (89·8%)	147/161 (91·3%)	129/146 (88·4%)	117/135 (86·7%)	80/102 (78·4%)	112/129 (86·8%)	106/121 (87·6%)	109/118 (92·4%)	115/132 (87·1%)	98/114 (86·0%)	110/116 (94·8%)	0·029	1291/1461 (88·4%)
Treatment episode (New/(New + Relapse))	172/187 (92·0%)	146/161 (90·7%)	135/146 (92·5%)	123/135 (91·1%)	94/102 (92·2%)	120/129 (93·0%)	116/121 (95·9%)	106/118 (89·8%)	125/132 (94·7%)	109/114 (95·6%)	111/116 (95·7%)	0·029	1357/1461 (92·9%)
Proportion of TB patients tested for HIV n/N (%)	1/187 (1·1%)	9/161 (5·6%)	35/146 (24·0%)	49/135 (36·3%)	62/102 (60·8%)	70/129 (54·3%)	31/121 (25·6%)	88/118 (74·6%)	108/132 (81·8%)	97/114 (85·1%)	102/116 (87·9%)	0·001	652/1461 (44·6%)
Proportion of TB patients with HIV results who had a positive HIV test n/N (%)	0/1 (0·0%)	1/9 (11·1%)	3/35 (8·6%)	9/49 (18·4%)	6/62 (9·7%)	9/70 (12·9%)	6/31 (19·4%)	9/88 (10·2%)	10/108 (9·3%)	12/97 (12·4%)	46/102 (45·1%)	<0·001	111/652 (17·0%)

**TB** = Tuberculosis; **%** = percent; **PTB** = Pulmonary TB; **EPTB** = Extra–Pulmonary TB; **new** = new TB disease patients; **relapse** = TB disease patients that relapsed. P = P–value is for temporal trend between 2005 and 2015, obtained using the Mann–Kendall test statistic for age but chi–squared test for trend for other variables.

### General population TB rates by age and year

**Fig 1:**
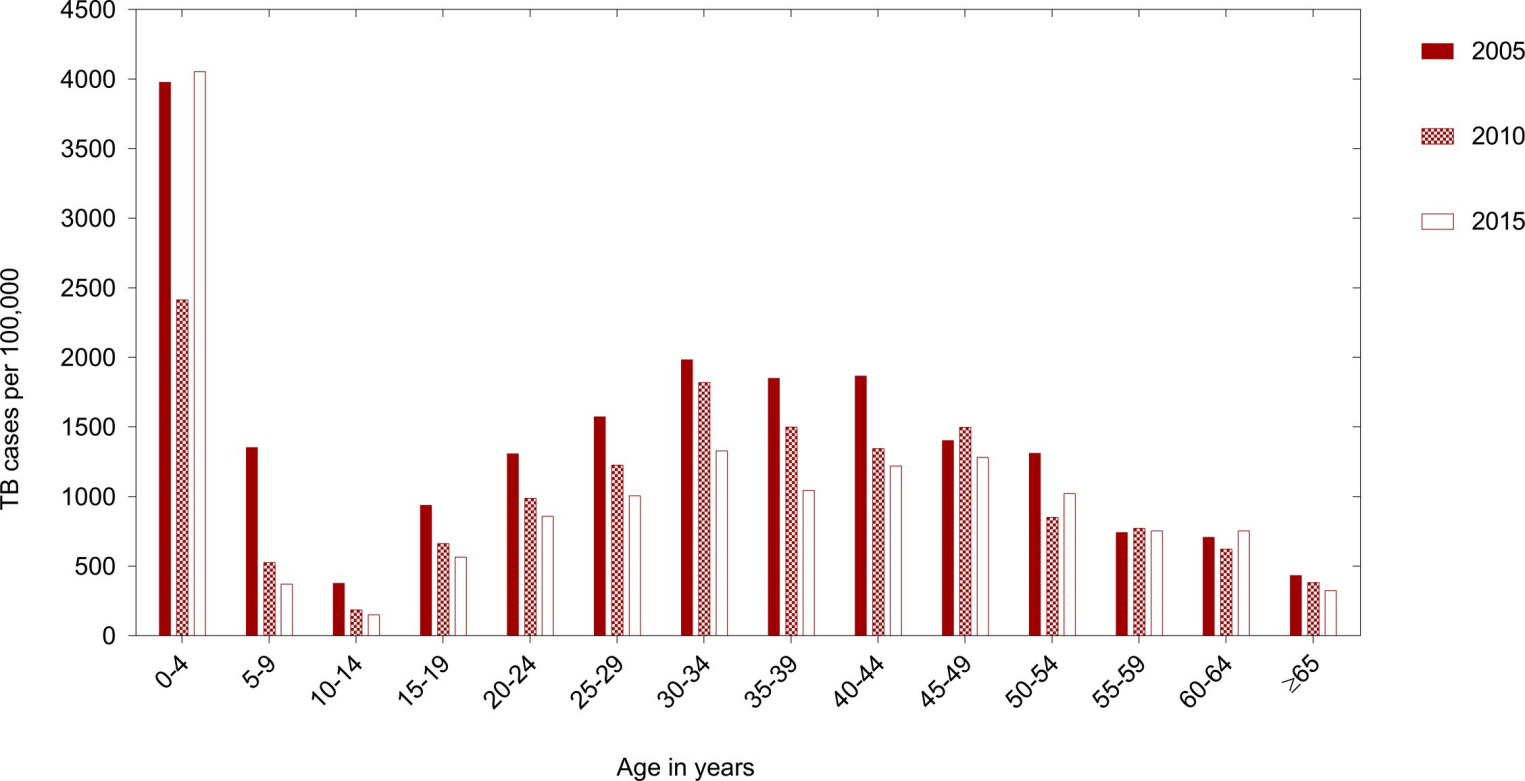
General population TB rates by age in 2005, 2010 and 2015. Legend: TB = Tuberculosis disease (all patients). TB rates reduced from 379 to 188 to 151 patients per 100,000 among adolescents aged 10–14 years old; and from 938 to 663 to 566 patients per 100,000 among adolescents aged 15–19 years old, in 2005, 2010 and 2015 respectively.

TB rates followed a bimodal age pattern with the highest risk in children aged below five years old (peak rate of 4,055 patients per 100,000), with a second peak among individuals aged 30–45 years (peak rate of 1,984 patients per 100,000). Adolescents aged 10–14 years had the lowest risk of TB disease (nadir rate of 151 patients per 100,000), with TB rates increasing in adolescence from 15–19 years of age onwards. Thereafter, TB rates declined consistently from age 45–49 years (Fig 1).

### Trends in childhood, adolescent and adult TB

**Fig 2:**
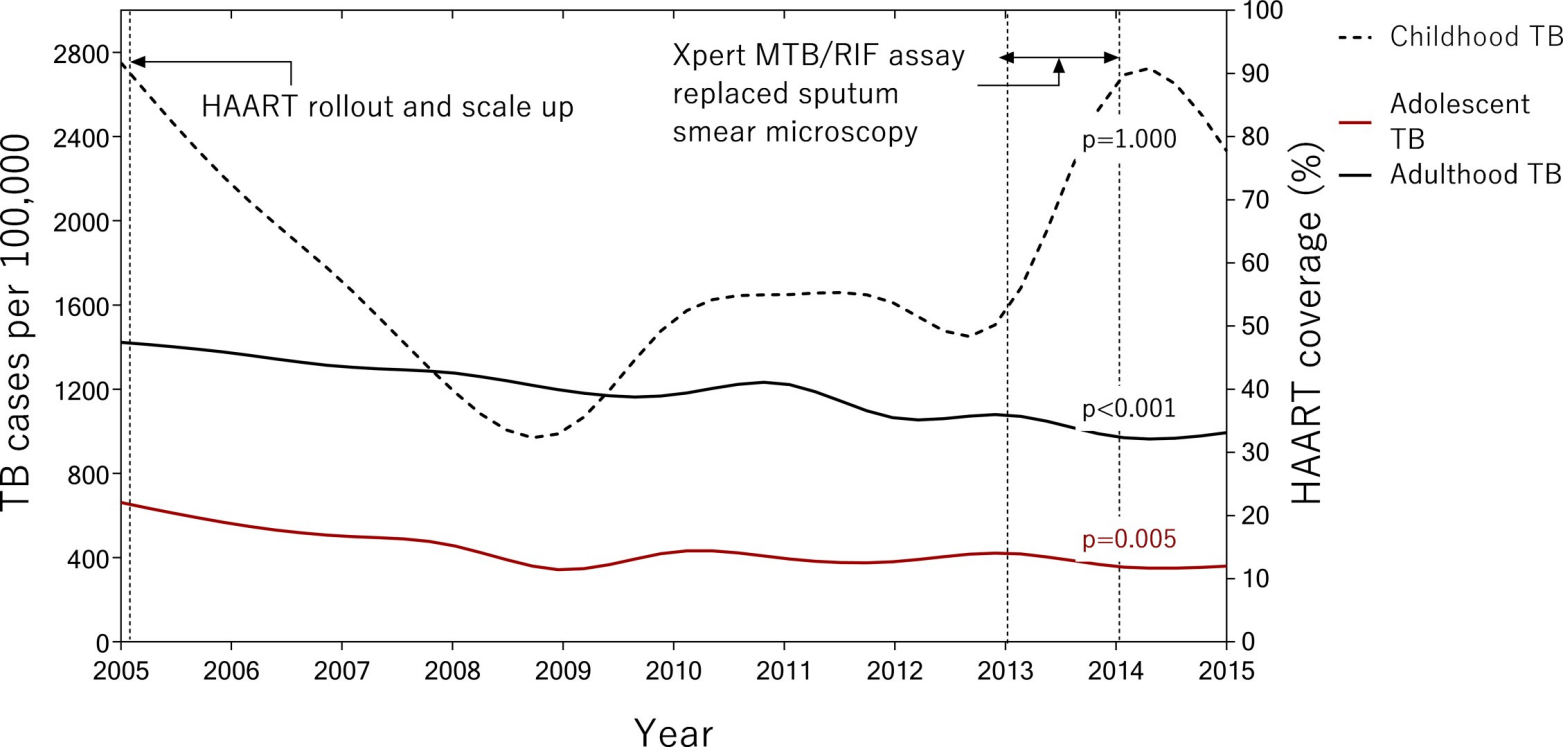
Comparison of adult and adolescent TB trends 2005–2015. Legend: TB = Tuberculosis disease (all TB). A = The period of rollout of Xpert MTB/RIF assay as a replacement for sputum smear microscopy. Between 2005–2015 all–TB rates declined by 15% from 2,750 to 2,331 per 100,000 among children; by 45% from 662 to 361 per 100,000 among adolescents; and by 30% from 1,423 to 994 per 100,000 among adults. P–value is for temporal trend between 2005–2015.

Average adolescent TB disease rates for the decade 2005–2015 were 477 patients per 100,000 and 361 patients per 100,000 for all–TB and microbiologically–confirmed TB, respectively. There was a statistically significant negative temporal trend in adolescent TB disease rates between 2005–2015 (per 100,000, TB disease rates declined by 45%; from 662 to 361; p = 0·005) see Fig 2 and Table 2), but that trend differed markedly pre–and post–2009. Among adolescents there was a statistically significant negative temporal trend in both all–TB (per 100,000, TB disease rates declined by 48%; from 662 to 343; p = 0·028) and microbiologically confirmed TB (per 100,000, TB disease rates declined by 49%; from 492 to 252; p = 0·027) for 2005–2009, which was not observed for the period 2009–2015 (rose 5%; from 343 to 361; p = 0·764 and rose 21%; from 252 to 305; p = 1·000, respectively). For comparison, between 2005–2015, adult all–TB rates declined by 30% from 1,423 to 994 per 100,000. The decline in adolescent TB vs. adult TB for 2005–2015 was not statistically significant. Childhood all–TB rates dropped 15% from 2,750 to 2,331 per 100,000 during the same period, but rebounded thereafter and consistently increased between 2009–2015 (see Fig 2).

**Table 2:** TB rate by year, age and diagnostic approach.

Year	Adolescent all–TB rates	Adolescent Annual change in TB rates (%)	Adolescent microbiologically confirmed TB rates	Overall TB rates	Overall Annual change in TB rates (%)	Overall microbiologically confirmed TB rates
2005	662	NA	492	1535	NA	890
2006	562	–15·1%	436	1376	–10·4%	817
2007	503	–10·5%	403	1237	–10·1%	785
2008	459	–8·7%	347	1113	–10·0%	714
2009	343	–25·3%	252	1002	–10·0%	659
2010	428	24·8%	345	1105	10·3%	657
2011	397	–7·2%	302	1155	4·5%	694
2012	382	–3·8%	320	1042	–9·8%	577
2013	422	10·5%	310	1050	0·8%	588
2014	359	–14·9%	258	1180	12·4%	551
2015	361	0·6%	305	1131	–4·2%	595
Period average	477	NA	361	1246	NA	720

**TB** = Tuberculosis; **TB rates** = TB notification rate per 100,000; **NA** = Not applicable. Period refers to 2005 to 2015. Overall refers to TB rates in the general population. Percentage annual change in TB rates varied between –25.3% and 24.8% and –10.4% and 12.4% among adolescents, and overall.

### Adolescent TB trends by age

**Fig 3:**
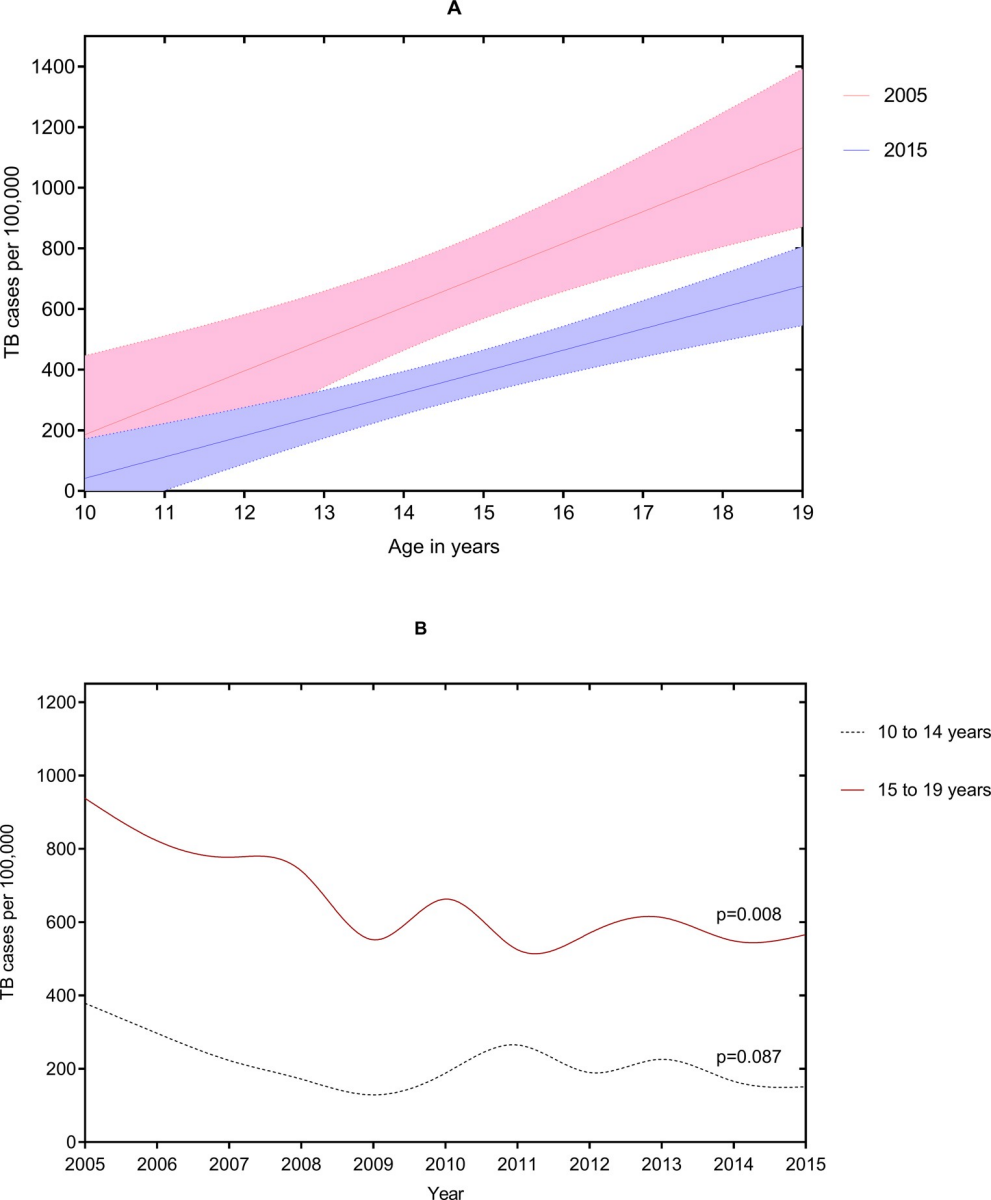
Adolescent TB temporal trends by age and year. Legend: TB = Tuberculosis disease (all–TB). **Panel 3A.** Adolescent TB disease rates by age and year. Between 2005–2015, TB rates declined by 63% (from 429 to 157 per 100,000; p–value for temporal trend (p–value) = 0·350) among adolescents aged 10 years old; by 29% (from 560 to 400 per 100,000; p–value = 0·640 among adolescents aged 15 years old; and by 40% (from 1120 to 677 per 100,000; p–value = 0·020) among adolescents aged 19 years old. **Panel 3B.** Adolescent TB disease rates by five–year age groups. TB rates declined by 60% (from 379 to 151 per 100,000) among adolescents aged 10–14 years old; and by 40% (from 938 to 566 per 100,000) among adolescents aged 15–19 years old. P–value is for temporal trend between 2005–2015.

Older adolescents contributed primarily to the overall reduction in adolescent TB disease rates (Fig 3A and Fig 3B). However, TB rates (per 100,000) declined statistically significantly in the period 2005–2009 among adolescents aged 10–14 years (declined by 66%; from 379 to 129; p–value 0·028) and those aged 15–19 years (declined by 41%; from 938 to 552; p–value 0·027).

### Adolescent TB trends by sex

**Fig 4:**
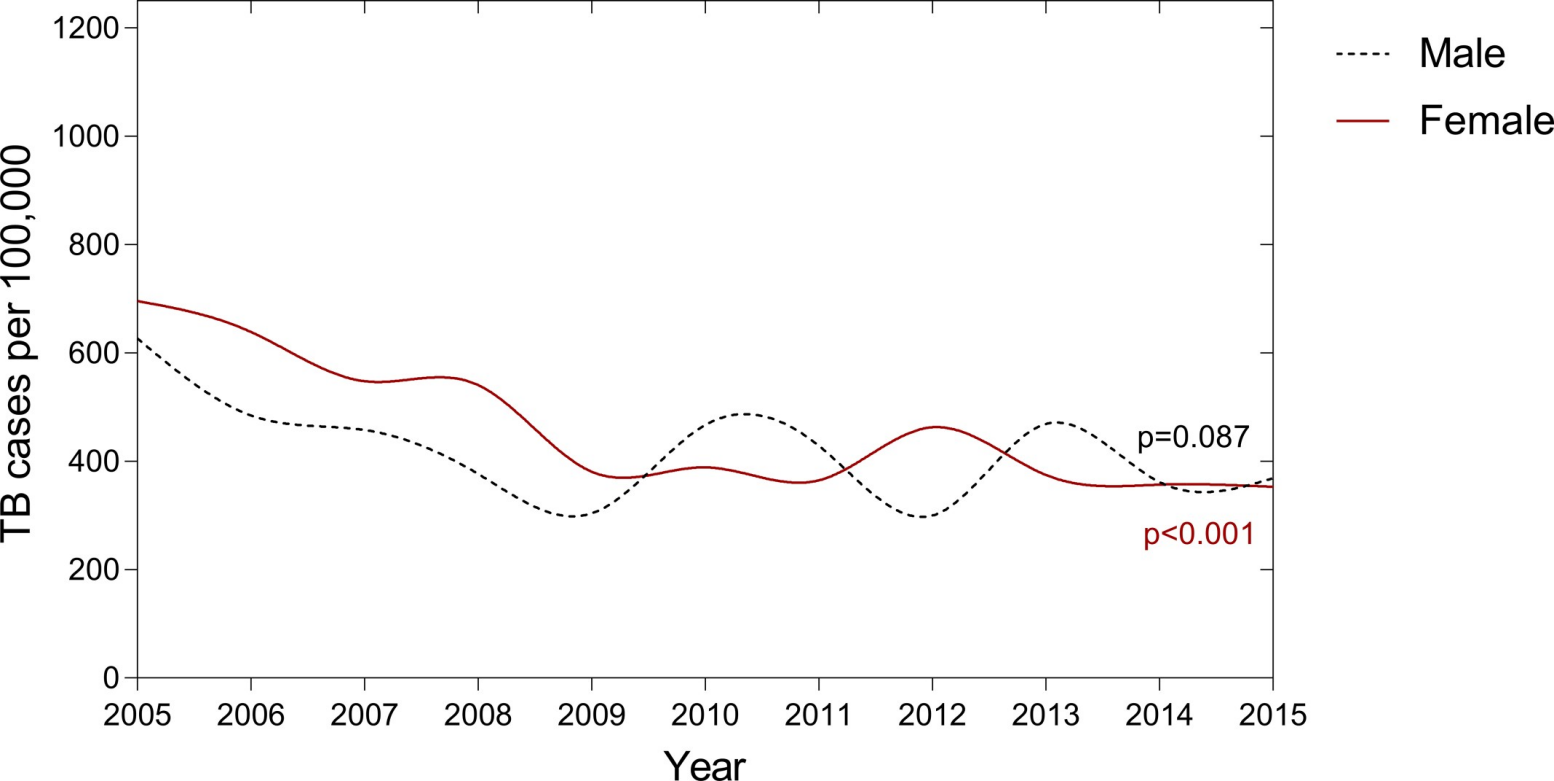
Adolescent TB temporal trends by sex. Legend: TB = Tuberculosis disease. Respectively among female and male adolescents, TB rates declined by 49% (from 696 to 353 patients per 100,000) and by 41% (from 627 to 369 patients per 100,000) from 2005 to 2015. P–value is for temporal trend between 2005 and 2015.

Average TB rates for the decade 2005–2015 were 425 patients per 100,000 among male adolescents and 496 patients per 100,000 among female adolescents. The temporal pattern of change in TB rates was similar by sex. Overall, a statistically significant temporal trend was only observed among female adolescents (see Fig 4) who had a higher baseline (2005) but a lower TB rate in 2015. TB rates among male adolescents declined by 41% (from 627 to 369; p–value 0·087) whereas among female adolescents TB rates declined by 49% (from 696 to 353; p–value <0·001). There was no statistically significant difference in adolescent TB notification rate by sex.

## Discussion

A significant fall in TB disease rates among adolescents in the Breede Valley sub–district, Western Cape Province, South Africa, was observed over the decade 2005–2015, although disease rates remain high. The percentage fall in adolescent TB disease rates was similar to the fall in adult TB disease rates; was more marked among older than younger adolescents; and occurred primarily during 2005–2009, but appeared to plateau thereafter in parallel with increased TB rates in younger children compatible with a period of sustained MTB transmission. The higher rates of TB notification observed in older adolescents (15–19 years) than younger adolescents (10–14 years) might also be explained by the differential ability to diagnose TB in these age groups: adolescents are in the transition from childhood (when diagnosis is harder due to clinical presentation of TB) to adulthood (when diagnosis is easier). There was no statistically significant difference in adolescent TB notification rate by sex, a finding similar to studies in South Africa[18] and the Philippines.[19]

The proportion of all TB occurring among adolescents statistically significantly reduced over this period, as it did among adults, whereas childhood TB proportionally increased. The median age of TB in the general population dropped slightly from 25 to 24 years of age. The lower median age of TB in the general population is unlike the shift towards older age of incident tuberculosis reported in Brazil,[20] which was due in part to population growth and aging, or that in Hong Kong due to immigration from higher TB burden regions of China.[21] An epidemiologic shift towards the elderly due to endogenous reactivation of remote TB infection is generally regarded as a sign of successful TB control in developed countries with low HIV burden.[22] In this case, the statistically significant fall in TB disease rates observed among adolescents in South Africa, who experience a very high force of TB infection, may be regarded as an initial sign of progress in TB control efforts.

Reported adolescent all–TB disease rates (both microbiologically–confirmed and clinically diagnosed) in high TB burden countries in Africa range between 180–679 per 100,000 (2002–2013):[23] 210–341 cases per 100,000 in three South African studies (2002–2013);[24]-[26] 180 cases per 100,000 in one Ugandan study (2009–2011);[27] and 679 cases per 100,000 in one Kenyan study (2010).[28] The relatively lower adolescent TB disease prevalence of 210 cases per 100,000 in the Marais *et al*. South African study[24] could be explained in part by the younger study participants (10–14 years) as compared to the other two South African studies that recruited adolescents aged 10–19 years old. The 2015 adolescent TB disease prevalence we report is similar to that published by Snow *et al*.[25] from South Africa (274 cases per 100,000), which could be explained in part by use of health system routine notification data in both studies.[25] TB notification data are thought to underestimate TB prevalence by at least 34% due to under–reporting and under–diagnosis.[29]

Low HIV testing rates between 2005–2010 likely underestimated the scale of HIV burden in adolescent TB patients, which was 45% in 2015. HIV co–infection impacts TB disease rates among older adolescents as HIV incidence increases with age,[30] for example between 30–50% of TB was attributable to HIV in older adolescents and adults in Kenya.[31] Since HIV increases TB risk up to 12–fold compared to that of HIV negative people,[32] the rapidly increasing population ART coverage from less than 1% in 2005 to 55% in 2015,[8] and the increasing threshold for ART initiation from a cut–off CD4 cell count of 200 cells/ml in 2004[33] to 350 cells/ml in 2012; and to 500 cells/ml in 2015[34] might be expected to contribute to a decline in both adult and older adolescent HIV–associated TB rates as observed in regional data[35], elsewhere in South Africa[1], and in mathematical modelling studies.[36] The impact of improved ART access could not be measured definitively for this adolescent population, due to lack of HIV incidence or prevalence data for this age group; this question requires modelling or further epidemiologic studies.

We note that adolescent TB rates declined substantially between 2005–2009 during nascent ART roll–out when HIV testing was less frequent; 21% (156/732) of adolescent TB notifications were HIV–tested, 12% (19/156) of whom were living with HIV. Adolescent all–TB rates prior to this period were 582 and 682 patients per 100,000 in 2003 and 2004 respectively (unpublished data from regional health authorities), suggesting that the onset of the fall in adolescent TB disease rates either occurred at the same time or preceded ART roll–out. However, we cannot confirm or exclude the possibility that ART access was responsible for the initial fall in adolescent TB disease rates, based on the available data. The biggest increase in population ART coverage occurred between 2010–2015, when adolescent and adult TB disease rates appeared to plateau and childhood TB rates increased, a pattern compatible with a period of sustained MTB transmission. A possible interpretation is that detection of childhood TB was improving at the same time as the benefits of ART roll-out for adolescents and adults might have been stabilising.

Socioeconomic factors affecting regional TB rates may also be at play. The proportion of informal dwellings in the study community increased from approximately 13% in 2005 to >22% in 2015.[15] It is conceivable that this worsening housing situation could have contributed to stagnation in adolescent TB disease rates between 2009–2015, but there were no notable changes in year–on–year migration patterns for seasonal agricultural work in this region.[37]

We have no evidence to believe that other changes in TB program activity or reporting impacted these observations. Adolescent TB treatment success rates remained stable and relatively high throughout the study period at an average of 84%; and the TB health management information system was strengthened between 2000–2005, when electronic notification of TB patients was introduced. Passive TB case–finding and treatment supported by DOTS was in place and remained virtually unchanged between 2005–2015. We believe our finding of declining adolescent TB temporal trends for 2005–2015 are generalizable to regions in South Africa with a similar TB epidemic and contextual socioeconomic status.

Key strengths of this analysis are the large dataset, high quality public laboratory support and robust time series analysis. However, TB notification underestimates true TB prevalence by at least 34% due to under–reporting and under–diagnosis.[29] Therefore, our results likely underestimate the true burden of TB among adolescents in South Africa. Other limitations include that HIV testing among adolescents was limited between 2005–2008; and data were lacking for adolescent HIV incidence, HIV prevalence, and ART usage, which limits our ability to determine whether the observed decrease in TB rates was driven by HIV–associated TB, HIV–negative TB, or both. The surveillance system does not record either ethnicity or socioeconomic information. We also acknowledge that our finding of declining adolescent TB disease rates might not be generalisable to other countries. Increasing temporal trends in TB disease rates in the general population have previously been noted in Botswana, Zimbabwe, Lesotho, South Africa and Swaziland[38] due to worsening HIV epidemic. Lastly, we cannot determine how accurately the changes in adolescent TB disease notification rate depict true changes in adolescent TB disease incidence rate.

In conclusion, the overall rate of reduction in adolescent TB over the decade 2005–2015 was almost two–fold that among adults and three–fold that among younger children. The substantial initial fall in adolescent TB disease notification rates between 2005–2009 plateaued thereafter, coupled with an increase in the TB notification rate among younger children that is consistent with a period of sustained MTB transmission. Given the apparent stagnation in adolescent TB and the marked increase in childhood TB rates after 2010, additional TB control measures, such as active case finding to reach the approximately 34% of individuals not captured by routine TB notification systems, and universal HIV testing of TB patients, are needed to sustain the encouraging early decline in TB disease. Further research using cohort study design to describe trends in force of *Mycobacterium tuberculosis* infection as part of close monitoring of the adolescent TB disease burden for timely policy action is needed to ensure that these early gains are consolidated.

## Supporting information

S1 File Data. (XLSX)
